# The burden of rotavirus gastroenteritis and hospital-acquired rotavirus gastroenteritis among children aged less than 6 years in Japan: a retrospective, multicenter epidemiological survey

**DOI:** 10.1186/1471-2431-13-83

**Published:** 2013-05-22

**Authors:** Hitoshi Tajiri, Yuriko Takeuchi, Tomoko Takano, Toshihiro Ohura, Ayano Inui, Kimie Yamamoto, Yoshihito Higashidate, Hisashi Kawashima, Shigeru Toyoda, Kosuke Ushijima, Gunasekaran Ramakrishnan, Mats Rosenlund, Katsiaryna Holl

**Affiliations:** 1 Osaka General Medical Center, 3-1-56, Mandai-Higashi, Sumiyoshi, Osaka 558-8558, Japan; 2 GlaxoSmithKline, Tokyo, Japan; 3 Sendai City Hospital, Sendai, Japan; 4 Saiseikai Yokohamashi Tobu Hospital, Saiseikai, Japan; 5 Kagoshima Medical Association Hospital, Kagoshima, Japan; 6 Sapporo Social Insurance General Hospital, Sapporo, Japan; 7 Tokyo Medical University Hospital, Tokyo, Japan; 8 Kanagawa Prefectural Shiomidai Hospital, Kanagawa, Japan; 9 Kurume University Medical Center, Kurume, Japan; 10 GlaxoSmithKline Vaccines, Wavre, Belgium; 11 Centre for Pharmacoepidemiology, Unit for Clinical Epidemiology, Department of Medicine, Karolinska Institutet, Karolinska, Sweden

**Keywords:** Children, Epidemiology, Gastroenteritis, Japan, Hospital-acquired, Rotavirus

## Abstract

**Background:**

Rotavirus is a leading worldwide cause of acute gastroenteritis in young children. This retrospective hospital-based study assessed the burden of rotavirus gastroenteritis in children younger than 6 years in Japan.

**Methods:**

Children admitted to eight hospitals for acute gastroenteritis between 2008 and 2009 were identified from hospital admission databases. Diagnosis of acute gastroenteritis/rotavirus gastroenteritis and hospital-acquired rotavirus gastroenteritis was confirmed based on either the International Classification of Diseases and Related Health Problems 10th revision (ICD10) codes (intestinal infectious diseases [AA00-AA09] and rotavirus gastroenteritis [A08.0]) or from rapid rotavirus diagnostic test results.

**Results:**

Of 13,767 hospitalized children, 11.9% (1,644), 4.8% (665) and 0.6% (81) were diagnosed with acute gastroenteritis, rotavirus gastroenteritis and hospital-acquired rotavirus gastroenteritis, respectively. Among acute gastroenteritis hospitalizations, 40.5% (665/1,644; ICD10 and rapid test) and 57.7% (645/1,118; rapid test only) were confirmed as rotavirus positive. Of 1,563 children with community-acquired acute gastroenteritis, 584 (37.4%) cases were confirmed as rotavirus positive. The median durations of hospitalization for all and community-acquired rotavirus gastroenteritis were 5.0 days (range: 2.0−133.0 days) and 5.0 days (range: 2.0-34.0 days), respectively. Among rotavirus gastroenteritis hospitalizations, 12.2% (81/665) of cases were hospital-acquired and the median duration of hospitalization was 10.0 days (range: 2.0-133.0 days). The median duration of additional hospitalization due to hospital-acquired rotavirus gastroenteritis was 3.0 days (range: 0–14 days). The overall incidence rate of hospital-acquired rotavirus gastroenteritis was 1.0 per 1,000 children hospital-days. The number of rotavirus gastroenteritis cases peaked between February and May in both 2008 and 2009, and the highest number of cases was reported in March 2008 (21.8%; 145/665). The highest number of rotavirus gastroenteritis hospitalizations (24.1%; 160/665) was observed in children aged 12–18 months. The proportion of hospital-acquired rotavirus gastroenteritis was higher in children aged below 18 months as compared to children at least 18 months of age (0.94 [95% CI: 0.71-1.21] vs. 0.39 [95% CI: 0.25-0.58]) and for children hospitalized for at least 5 days compared to those hospitalized for less than 5 days (0.91 [95% CI: 0.72-1.14] vs. 0.15 [95% CI: 0.05-0.32]).

**Conclusions:**

Both community- and hospital-acquired rotavirus gastroenteritis are significant public health problems in Japan. Data from this study justify the need for the introduction and implementation of rotavirus vaccination in the Japanese national immunization program.

**Trial registration:**

ClinicalTrials.gov, NCT01202201

## Background

Rotavirus (RV) is the most common etiological cause of acute gastroenteritis (GE) resulting in hospitalization among young children throughout the world [1]. In Japan, the burden of RVGE disease is substantial, with approximately 800,000 children aged below 6 years visiting clinics and hospital outpatient departments annually. A previous study conducted in Japan reported an overall incidence of outpatient visits of 11 cases per 100 person-years due to RVGE in children less than 6 years of age; the highest incidence (27 cases per 100 person-years) was observed in infants [2]. In another single-center study conducted between 2008 and 2010 in Kyoto, the proportion of children aged below 5 years diagnosed with RVGE was reported to be 56% [3]. Although episodes of RVGE can potentially be severe [4], due to a low mortality rate associated with RVGE in Japan, local awareness of the disease is low. Nevertheless, in Japan, RVGE is a common cause of acute GE hospitalizations whether community- or hospital-acquired and results in significant clinical and economic burden [5].

There are no official, evidence-based guidelines on the treatment of pediatric acute GE in Japan [6], but RVGE is most commonly managed using intravenous fluid replacement therapy [7]. Prophylactic interventions, such as RV vaccination, may represent an effective primary public health strategy to reduce the burden of RVGE [8]. Two orally administered RV vaccines, which have demonstrated good efficacy and safety profiles in global clinical trials, have been licensed in most countries: *Rotarix*™ (GlaxoSmithKline Vaccines, Belgium) and *RotaTeq*® (Merck and Co., Inc., Whitehouse Station, NJ, USA) [8,9]. In 2009, the World Health Organization (WHO) recommended the inclusion of RV vaccines in the routine childhood vaccine programs of all countries [9].

The objectives of this study were to estimate the burden of overall acute GE, RVGE and hospital-acquired RVGE in hospitalized children younger than 6 years of age, as well as to assess the age and seasonal distributions; and duration of hospitalization and additional hospitalization associated with hospital-acquired RVGE. Our findings could have important implications for the introduction of routine RV vaccination into the Japanese national immunization program. Furthermore, up to-date epidemiological data on RVGE disease may help evaluate the impact and cost-effectiveness of RV vaccination programs in Japan.

## Methods

### Study design

**Figure 1:**
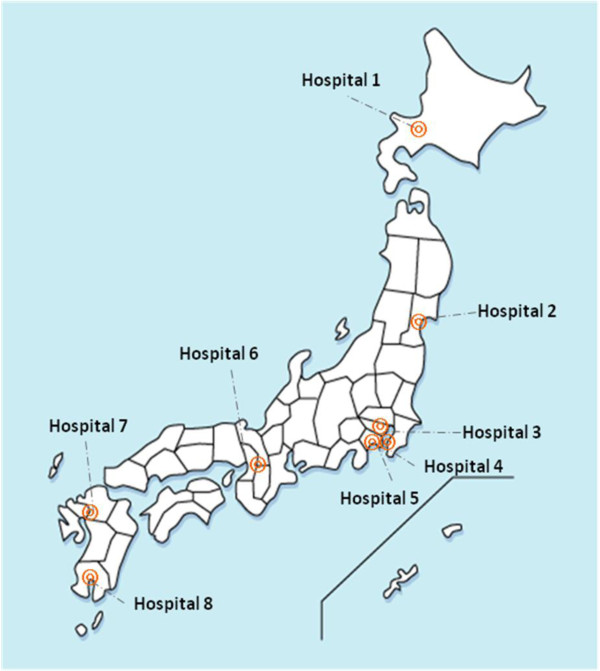
Geographic distribution of the participating hospitals, 2008–09.

This retrospective, multi-center epidemiological study was conducted between January 2008 and December 2009 at eight regional core hospitals in the urban areas of Japan, which represented an optimum geographic coverage (Figure 1). All study hospitals treated pediatric infectious diseases including acute GE, and were included if they had at least 15 pediatric inpatient beds (range in the study hospitals 16–50) and routinely tested for the presence of RV using rapid tests. At each hospital, one nurse was allocated to either seven or ten children and participating hospitals provided primary, secondary and tertiary emergency care for children [10].

Children aged less than 6 years, who were hospitalized for acute GE or RVGE during the survey period, were included in the study. These children were identified through the electronic hospital databases. Diagnosis of acute GE/RVGE and hospital-acquired RVGE was confirmed either according to the International Classification of Diseases and Related Health Problems 10th revision (ICD 10) codes (intestinal infectious diseases [AA00-AA09] and RVGE [A08.0]) or based on the results from rapid RV diagnostic tests, using immune-chromatography (*RapidTesta*®ROTA-ADENO, Orion Diagnostica; Bioline SD Rota/Adeno Rapid, Standard Diagnostics, INC, Kyonggi, Korea; *BD Rota/Adeno Examan*™ stick, Becton, Dickinson & Co; and *Premier*™ *Rotaclone*®, Meridian Bioscience, Inc, Ohio, USA).

Acute GE was based on the ICD 10 codes for intestinal infectious diseases: with occurrence of diarrhea for less than 14 days. Community-acquired RVGE was defined as RVGE hospitalization other than hospital-acquired RVGE. Hospital-acquired RVGE was defined as occurrence of RVGE at least 48 hours after hospital admission or within 48 hours after hospital discharge. For a child who was hospitalized for acute GE within the last 48 hours investigators ascertained the presence of RV from the medical chart. RV tests were not performed in hospitals for subjects who had already been diagnosed with RVGE at other clinics or hospitals, since the variance of diagnosis intensity depended on subjects and not on the participating hospitals.

Medical histories were collected from medical charts or the hospital database, and the duration of hospitalization for acute GE and community-acquired RVGE, define as the number of days between the date of admission and date of discharge, was automatically calculated in the medical chart database. The duration of additional hospitalization for hospital-acquired RVGE (in days) was estimated as the difference between the usual average number of days spent in hospital due to the specific medical condition and the number of days the child actually spent in hospital.

This study was conducted in compliance with Good Clinical Practice (GCP) guidelines and the Japanese Ministry of Health, Labor and Welfare’s (MHLW) ethical guidelines for epidemiological research. Since this was an observational survey using hospital database data, informed consent was not obtained from the enrolled subjects. This is in accordance with the Japanese ethical guidelines for epidemiological research. All the relevant documents were reviewed by the ethical committees and ethical approval was sought from each of the participating hospitals.

### Endpoints and statistical analysis

All analyses were descriptive and the demographic distribution of children aged below 6 years who were hospitalized with acute GE, RVGE and hospital-acquired RVGE were presented. The overall proportions of acute GE, RVGE and hospital-acquired RVGE among all the subjects and proportions of hospital-acquired RVGE stratified by hospital and patient characteristics were estimated with 95% confidence intervals (CI) using the Exact Method [11]. Median duration of hospitalization (in days) due to RVGE, community-acquired RVGE and hospital-acquired RVGE, and the median duration of additional hospitalization due to hospital-acquired RVGE were estimated. The incidence of hospital-acquired RVGE was calculated by dividing the number of hospital-acquired RVGE cases by the total number of hospital days among all at-risk children and was expressed per 1000 hospital days. When estimating incidence, 95% CI was calculated using Poisson’s method. All statistical analyses were performed using SAS version 9.2.

## Results

Among 13,767 hospitalized children, 1,648 were diagnosed with acute GE and were included in the study, of whom four were excluded from the final analysis as they did not meet the inclusion criteria. The demographic characteristics of the 1,644 (11.9%) children included in the final analysis are shown in Table 1.

**Table 1:** Demographic characteristics of children aged less than 6 years, hospitalized in Japan during 2008–09

**Subject group**		**Acute GE**^**2 **^**(N =1644)**	**RVGE (N=665)**	**Hospital-acquired RVGE (N=81)**
**Gender (male/female) (%)**		57.0/43.0	54.6/45.4	61.7/38.3
**Age (months)**	**Mean ± SD**^**1**^	24.6 ± 18.07	23.1 ± 15.88	15.9 ± 13.84
	**Median (range)**	19 (0–71)	18 (0–71)	13 (0–62)
**Underlying medical condition n (%)**		10 (0.6)	5 (0.8)	0 (0.0)
**-Cardiac disease**		14 (0.9)	2 (0.3)	1 (1.2)
**- Gastrointestinal disease**		20 (1.2)	8 (1.2)	3 (3.7)
**-Neurological disease**		15 (0.9)	6 (0.9)	2 (2.5)
**-Prematurity**		46 (2.8)	18 (2.7)	8*(9.9)
**-Pulmonary disease**		54 (3.3)	20 (3.0)	8 (9.9)
**-Others**		1485 (90.3)	606 (91.1)	59 (72.8)
**- No condition**				

**Note: **^1^Standard deviation; ^2^Acute gastroenteritis; *Information on hospitalization duration missing for one case.

Among the 1,644 children with acute GE, 665 (40.5%) were confirmed with RV, using either ICD 10 diagnostic codes or RV diagnostic tests. The proportion of overall RVGE among all hospitalized children aged less than 6 years was therefore 4.8% (665/13,767). RV diagnostic testing, performed on stool samples from 1,118 children (68.0%) with acute GE, confirmed that 645 (57.7%) were RV-positive. Of the 1,563 children with community-acquired acute GE, 584 (37.4%) were RV-positive. Eighty-one of the 665 RV-positive cases, (12.2%) were confirmed as hospital-acquired RVGE. Similar proportions of RVGE (overall and hospital-acquired) were observed in children aged less than 5 years (Table 2). Underlying medical conditions were present in 159 cases (9.7%) of acute GE hospitalizations, 59 cases (8.9%) of RVGE hospitalizations and 22 cases (27.2%) of hospital-acquired RVGE cases (Table 1).

**Table 2:** Proportion of RVGE diagnosed in hospitalized children (ICD10 codes and RV rapid test kits) in Japan during 2008–09

**Age group**	**Proportion of RVGE among all hospitalizations (n/N; 95% CI)**	Proportion of RVGE among all acute GE hospitalizations (n/N; 95% CI)*		**Proportion of hospital-acquired RVGE among RVGE (n/N; 95% CI)**
		**Based on ICD code and kit results**	**Based on kit test results**	
**<2 years**^**1**^	4.9% (409/8285; 4.5−5.4)	41.2% (409/993; 38.1−44.3)	55.0% (398/723; 51.3−58.7)	15.2% (62/409; 11.8−19.0)
**2−<4years**	5.8% (191/3265; 5.1−6.7)	47.6% (191/401; 42.6−52.6)	69.7% (186/267; 63.8−75.1)	7.9% (15/191; 95% CI: 4.5−12.6)
**4−<6 years**	2.9% (65/2213; 2.3−3.7)	26.0% (65/250; 20.7−31.9)	47.2% (60/127; 38.3−56.3)	6.2% (4/65; 95% CI: 1.7−15.0)
**<5 years**^**2**^	5.0% (643/12771; 4.7−5.4)	41.7% (643/1542; 39.2−44.2)	58.3% (625/1072; 55.3−61.3)	12.4% (80/643; 10.0−15.2)
**<6 years**^**3**^	4.8% (665/13767; 4.5−5.2)	40.5% (665/1644; 38.1−42.9)	57.7% (645/1118; 54.7−60.6)	12.2% (81/665; 9.8−14.9)

**Note: **^1^<2 is due to the highest disease burden in this age group; ^2^<5 is to make it possible to compare with the result from other developed countries; ^3^<6 years is to align with Japanese schooling system (children start elementary education at the age of 6 years).

*Both indicate the same proportion.

**Figure 2:**
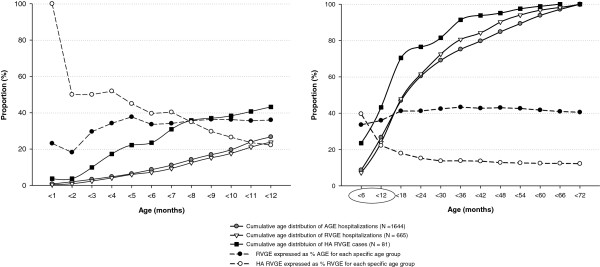
Age distribution of acute GE, RVGE hospitalizations and hospital-acquired RVGE cases.

Cumulative age distributions of acute GE and RVGE hospitalizations are shown in Figure 2. Three cases of RVGE were reported in infants aged below 1 month. The highest proportion of acute GE and RVGE hospitalizations were observed in children aged 12–18 months (20.2% [332/1,644] and 24.1% [160/665], respectively), followed by children aged 6–12 months (18.0% [296/1,644] and 16.6% [110/665], respectively).

The median duration of hospitalization for all RVGE and community-acquired RVGE was 5.0 days (range: 2.0−133.0 days) and 5.0 days (range: 2.0−34.0 days). The longest duration of hospitalization due to all RVGE (median 9.0 days [range: 5.0−108.0 days]) was observed in children aged 5–6 months (n/N= 7/665).

The number of RVGE cases peaked between February and May in both 2008 and 2009 and the highest number of cases (21.8%; 145/665) was recorded in March 2008.

Among the children aged below 6 years who were hospitalized, the overall proportion of hospital-acquired RVGE was estimated to be 0.6% (N=81; 95% CI: 0.5−0.7) (0.9% [95% CI: 0.7−1.1] in 2008 and 0.3% [95% CI: 0.2−0.5] in 2009). Across the hospitals, the proportions of hospital-acquired RVGE ranged from 0.1% (95% CI: 0.0−0.7) to 1.7% (95% CI: 1.1−2.6). The overall incidence of hospital-acquired RVGE was 1.0 per 1,000 person-days (95% CI: 0.8−1.2) in children aged less than 6 years (1.4 [95% CI: 1.1−1.8] in 2008 and 0.5 [95% CI: 0.3−0.8] in 2009). The highest number of hospital-acquired RVGE cases was observed in children aged 12–18 months (27.2%; 22/81), followed by those aged 0–6 months (23.4%; 19/81) and 6–12 months (19.7%; 16/81) (Figure 2). All three cases of RVGE in infants aged below 1 month and 50.0% (3/6) of cases in children aged less than 2 months were hospital-acquired. A decreasing trend of hospital-acquired RVGE among RVGE hospitalizations with age was observed, with higher proportions being reported in younger children (especially in infants aged below 1 month) (Figure 2).

The median duration of hospitalization for hospital-acquired RVGE cases was 10.0 days (range: 2.0−133.0), but varied according to the presence of different medical conditions: gastrointestinal disease (26.0 days [range: 26.0−26.0]); neurological disease (108.0 days [range: 23.0−133.0]); prematurity (31.0 days [25.0−37.0]); pulmonary disease (10.0 days [range: 7.0−15.0]); and others (20.5 days [range: 12.0−89.0]). In subjects without any underlying medical condition, the median duration was 9.0 days (range: 2.0−43.0). The median duration of hospitalization for children with hospital-acquired RVGE also varied by hospital and ranged from 6.5 days (range: 4.0−15.0) to 20.5 days (range: 15.0−26.0). The median duration of additional hospitalization as a result of hospital-acquired RVGE was 3.0 days (range: 0.0–14.0).

The proportion of hospital-acquired RVGE was higher in children aged below 18 months as compared to children at least 18 months of age (0.94 [95% CI: 0.71−1.21] vs. 0.39 [95% CI: 0.25−0.58]) and for children hospitalized for at least 5 days compared to those hospitalized for less than 5 days (0.91 [95% CI: 0.72−1.14] vs. 0.15 [95% CI: 0.05−0.32]). The proportions of hospital-acquired RVGE were also estimated to be higher in hospitals: with an allocation of one nurse per seven patients compared to those with an allocation of one nurse per ten patients (0.74 [95% CI: 0.56−0.95] vs. 0.18 [95% CI: 0.04−0.54]); with at least 30 beds compared to fewer than <30 beds (0.74 [95% CI: 0.58−0.92] vs. 0.53 [95% CI: 0.34−0.79]); and in hospitals with an isolation ward (0.75 [0.59−0.94] vs. 0.24 [95% CI: 0.08−0.56]) compared to hospitals without an isolation ward) (Table 3). Among all the children with RVGE and an underlying medical condition 37.3% (22/59) cases were hospital-acquired RVGE compared to only 9.7% (59/606) of cases in children who did not have any underlying medical condition.

**Table 3:** Characteristics of hospital-acquired RVGE in children aged below 6 years in Japan during 2008–09 (N=13767)

**Characteristics**	**Categories**	**N**	**n**	**% (95% CI)**
**Age at the time of hospitalization**	≥18 months	6125	24	0.39 (0.25−0.58)
	<18 months	6075	57	0.94 (0.71−1.21)
**Hospitalization duration**^**1**^	≥5 days	8128	74	0.91 (0.72−1.14)
	<5 days	4053	6	0.15 (0.05−0.32)
	missing	19	1	5.26 (0.13−26.03)
**Nurse allocation**^**┼**^	N* =7	10573	78	0.74 (0.58−0.92)
	N* =10	1627	3	0.18 (0.04−0.54)
**Number of beds**^**┼**^	≥30	7854	58	0.74 (0.56−0.95)
	<30	4346	23	0.53 (0.34−0.79)
**Status of hospital with isolation ward/room**^**┼**^	With	10122	76	0.75 (0.59−0.94)
	Without	2078	5	0.24 (0.08−0.56)

^1^Cut-off point of 5 days was decided based on mean hospitalization duration in Japanese infectious pediatric wards (4.7 days for children aged 1–4 years) [21].

Note: *1 nurse per 7/10 patients; N: number of all hospitalizations <6 years of age in the given category who are at risk of acquiring hospital-acquired RVGE; n: number of hospital-acquired RVGE subjects.

^**┼**^Hospital data: Number of hospitals with N*=7: 6 hospitals and N*=10: 2 hospitals; Number of hospitals with ≥30 beds and <30 beds: 4 hospitals; Number of hospitals with isolation ward/room: 6 hospitals and without an isolation ward/room: 2 hospitals.

## Discussion

The present study is one of the largest multicenter hospital-based studies conducted in Japan that describes the burden of RVGE disease, including hospital-acquired RVGE in pre-school children. The findings of this study estimate that 4.8% of all hospitalizations in children aged below 6 years are due to RV. Despite the retrospective study design, 68.0% of hospitalizations due to acute GE were tested for the presence of RV of which 57.7% cases were RV-positive with the median duration of hospitalization of 5.0 days. These observations are consistent with previously published data on RVGE from Japan [7]. In this study, the highest burden of hospitalized RVGE disease was observed in children younger than 2 years of age, which is similar to that reported from several observational studies conducted in Europe [12-15]. The occurrence of RVGE usually exhibits a seasonal pattern [16] and in this study, a peak in the number of RVGE cases was observed between February and May in both 2008 and 2009. The highest number of RVGE cases was observed in March, suggesting that in line with previous reports, RVGE is a common disease in Japan until early summer and is no longer winter-specific [17,18].

We found that the proportion of hospital-acquired RVGE among all hospitalizations of children aged less than 6 years was 0.6% and was associated with median duration of additional hospitalization of 3 days. This proportion was well within the range that was observed in children below 5 years in the European Union (0.3−27.7%) [19]. We believe that this relatively low proportion of hospital-acquired RVGE could be due to different infection control procedures in the Japanese healthcare system [20]. However, there are no differences between Japanese and European hospitals and the reason for this apparent difference remains to be unknown. The incidence of hospital-acquired RVGE of 1.0 per 1,000 hospital-days reported in our study was well within the range (0.0−1.87 per 1,000 days of hospitalization) reported in Europe by Forster et al. [13]. In other observational studies conducted across Europe, it was reported that 95.7% of all hospital-acquired RVGE cases occurred in children younger than 2 years of age [13]. Our results also recorded higher proportions of hospital-acquired RVGE in children below 2 years of age compared with older children. The median duration of addition hospitalization due to hospital-acquired RVGE of 3.0 days which we observed was slightly lower than the mean duration of hospitalization (4.7 days) in infectious disease wards among children aged 1–4 years in Japan [21].

This study provides signs of patient and hospital characteristics that may be associated with the occurrence of hospital-acquired RVGE. Patient characteristics such as duration of hospitalization, age, prematurity or low birth-weight, severe immuno-deficiencies and absence/presence of underlying medical conditions have been previously reported as risk factors for hospital-acquired RV infection [19]. Indeed, in our study children with underlying medical conditions had a higher proportion of hospital-acquired RVGE compared to children without these conditions. We found higher proportions of hospital-acquired RVGE in children aged below 18 months and in those who were hospitalized for at least 5 days compared to those aged at least 18 months or hospitalized for fewer than 5 days, respectively. Children younger than 18 months of age may be more susceptible to hospital-acquired RVGE as well as need more attention from caregivers and may therefore be more likely to acquire RV infection from them. The proportions of hospital-acquired RVGE were higher in children at hospitals with a nurse allocation of one per seven patients than at hospitals with an allocation of one per ten patients, and also in hospitals with an isolation ward/room. The observation associated with higher nurse allocation may be related to the fact that nurses may be considered as reservoirs of RV infection. However, this study was not designed specifically to study the association of hospital characteristics and occurrence of hospital-acquired RVGE, presented in previous studies [19], therefore the clinical implications of these data need to be interpreted with caution.

The direct medical cost of a single hospitalization with a mean duration of hospitalization of 5.4 days due to RVGE in Japan has been estimated to be 1,888 US$ (221,000 JPY; 1 US$ = 83.6 JPY [22]) [3]. Considering the median duration of a single RVGE hospitalization of 5.0 days in this study, the direct medical cost per RVGE case per day is estimated to be 378 US$. Accordingly, the direct medical cost for an additional 3.0 days of hospitalization due to hospital-acquired RVGE in this study is estimated to be 875 US$ per hospital-acquired RVGE case. By extrapolating data from Nakagomi et al. [7], 78,000 RVGE hospitalizations were estimated in Japan per year among children aged below 5 years, of which 12.4% (9,672/78,000) could be considered as hospital-acquired RVGE cases per year. Therefore, the nationwide cost for hospital-acquired RV infection is estimated to be as high as 8,466,341 US$ per year in Japan.

There are several reasons why the results of this study may be applied to the general Japanese pediatric population below 6 years of age hospitalized for acute GE. This is the largest multi-hospital study yet conducted, including 8 hospitals across Japan capturing ~0.5% of the total RVGE hospitalizations that are expected to be reported in children younger than 6 years of age every year [7]. In addition, this study obtained laboratory confirmation using rapid RV diagnostic testing for 68.0% of all acute GE cases, which minimized the possible misclassification of RVGE. The data from this study allow for real world estimates of hospital-acquired RVGE and the duration of additional hospitalization due to hospital-acquired RVGE. Indeed, the hospital databases used in this study confirmed the good quality and diagnostic accuracy. Moreover, the data reported here are consistent with previous studies [3,7,23].

Besides the inevitable limitations of all retrospective surveillance studies [24], several specific limitations also need to be considered when interpreting the results of this study. Some of the RVGE cases did not have a RV rapid test recorded in the medical chart (~32%), which we believe was due to the fact that testing for the presence of RV may have been conducted previously in other clinics or hospitals. However, these subjects had the specified ICD 10 code for RVGE recorded in hospital database. Furthermore, the number of hospital-acquired RVGE cases may have been underestimated in this study due to missing ICD 10 codes for some RVGE cases and the difficulty of capturing data on the onset of hospital-acquired RVGE within 48 hours of discharge from hospital, unless the children returned to the participating hospitals for subsequent treatment. Since this was a retrospective database study, no diagnostic criteria were used as confirmation of diagnosis. The diagnosis was based on clinical practice and results of the RV diagnostic rapid tests were taken from the medical charts. Our method of determining median duration of additional hospitalization for hospital-acquired RVGE did not take into account the potential impact of accompanying chronic medical conditions and this may thus limit the generalizability of our findings. Another limitation of the present analysis is that although subjective, the duration of additional hospitalization for hospital-acquired RVGE was estimated based on careful examination of the medical chart and prescription record by comparing the duration of hospitalization for the specific case and the usual average duration of hospitalization for certain diseases if not associated with RVGE. However, duration of additional hospitalization was estimated as zero if medical chart review revealed that RV was unlikely to have had any impact on hospitalization duration (e.g. duration of hospitalization due to primary diagnoses was too long and hospital-acquired RVGE occurred in the middle of hospitalization for initial diagnoses etc.). Finally, the incidence calculated in this study may have been underestimated since the incubation period for hospital-acquired RV infection was not taken into account.

## Conclusions

The results of this large, multicenter study conducted in Japanese hospitals suggest that RV is a major cause of acute GE disease in children younger than 6 years of age and leads to a substantial burden on the public healthcare system in Japan. These results warrant further investigation into the need for implementing RV vaccination into the national immunization program of Japan. The baseline data from this study will be useful when assessing the impact and cost-effectiveness of future RV vaccination programs.

## Abbreviations

RV: Rotavirus; GE: Gastroenteritis; ICD 10: International Classification of Diseases and Related Health Problems 10^th^ revision; CI: Confidence interval.

## Competing interests

Hitoshi Tajiri, Toshihiro Ohura, Hisashi Kawashima, Kosuke Ushijima, Tomoko Takano and Shigeru Toyoda received institutional grants and consultation fees from GlaxoSmithKline group of companies for this study. Ayano Inui, Kimie Yamamoto, Yoshihito Higashidate, received institutional grants from GlaxoSmithKline group of companies for this study. Shigeru Toyoda received a lecture fee from the sponsoring company for activities outside this current publication. Katsiaryna Holl and Gunasekaran Ramakrishnan are employees of GlaxoSmithKline group of companies. Mats Rosenlund and Yuriko Takeuchi were employees of GlaxoSmithKline group of companies and GlaxoSmithKline Japan, respectively at the commencement of this study.

## Authors’ contributions

HT, YT, TT, TO, AI, KY, YH, HK, ST, KU, MR and KH took part in either the conception and design of the study, protocol development, study results analysis and interpretation and/or collection of the data. GR performed the statistical data analyses. All authors reviewed and commented on the draft manuscript, and all authors read and approved the final manuscript.

## Pre-publication history

The pre-publication history for this paper can be accessed here:

http://www.biomedcentral.com/1471-2431/13/83/prepub

## Trademarks

*Rotarix* is a registered trademark of GlaxoSmithKline group of companies.

*RotaTeq* is a registered trademark of Merck and Co., Inc., USA.

*RapidTesta* Rota Adeno is a registered product of SEKISUI MEDICAL CO., LTD. Immunocard ST Rotavirus is manufactured by TFB, INC.

BD Rota/Adeno stick is manufactured by Becton, Dickinson & Co.
